# Is a Large Neck Circumference a Predictive Factor for Poor Semen Quality in the Turkish Population?

**DOI:** 10.1007/s43032-025-01850-6

**Published:** 2025-03-26

**Authors:** Duygu Dursunoglu

**Affiliations:** Department of Histology-Embryology and IVF Clinic, Faculty of Medicine, Selcuk University, Konya, Turkey

**Keywords:** Male infertility, Semen quality, Central obesity indices, Neck circumference, Body mass index

## Abstract

Currently, the potential role of obesity on semen quality remains unclear. In recent years, alternative anthropometric indices to body mass index (BMI), which can precisely distinguish body fat distribution, have been described to estimate central adiposity. However, the role of neck circumference (NC), a novel index of central adiposity, on semen quality is unknown. This study aims to reveal the potentials of adiposity indices, including NC, in predicting poor semen quality. A total of 4739 male participants between the ages of 17–55 were included in this cross-sectional study. Semen parameters, including sperm concentration, total count, total, progressive and rapid progressive motility and vitality, were divided into two categories according to the WHO classification for male infertility. As anthropometric measurements of obesity, BMI and central obesity indices including waist circumference (WC), hip circumference (HC), waist-to-hip ratio (WHpR), waist-to-height ratio (WHtR) and NC were examined. The predictive potentials of obesity indices for low semen parameters were evaluated by Receiver Operating Characteristics (ROC) curve analysis. The associations of obesity indices with semen parameters were analyzed by binary logistic regression analyze after adjusting potential confounding factors. Patients with lower semen parameters had higher obesity indices than those with normal parameters. All obesity indices have the predictive potentials for low semen parameters. After adjustment for confounders, the strongest associations were found between HC with sperm count parameters, WC and WHtR with sperm vitality and motility parameters and WHpR and NC with rapid progressive motility. Obesity plays an important role in male infertility. Body fat distribution appears to have specific roles on sperm functionality, which may influence different infertility markers. NC is a strong predictor for sperm rapid progressive motility, suggesting a role for male infertility.

## Indroduction

Infertility is an important reproductive health problem affecting approximately 15% of couples worldwide [1]. The male factor is involved in 50% of infertility cases and alone contributes to approximately 25–30% of cases [2]. Obesity is a global health problem that has become increasingly common in recent years [3]. The decline in semen quality concurrent with the increasing prevalence of obesity suggests the potential role of obesity in male infertility [4]. Moreover, increasing evidence shows that male infertility is associated with an increased risk of many health conditions, including cardiovascular, metabolic, oncological, and autoimmune diseases, and that male infertility may be a predictor of overall health status [5]. Obesity may affect male fertility through multiple pathways, including erectile dysfunction, hormonal imbalance, reduced semen quality, increased production of adipokines, inflammatory mediators and reactive oxygen species (ROS), epigenetic modifications and elevated testicular temperature [6–8].

Obesity is characterized by excess fat accumulation, which has a negative impact on many health conditions, and is clinically defined by body mass index (BMI) [9]. However, it is well known that body fat distribution plays a more important role than its amount in the development of obesity-related complications and that excess visceral fat leads to more serious consequences [10]. BMI does not distinguish between lean and fat body mass and is not sufficient to assess body fat distribution. Therefore, BMI has low sensitivity in predicting central adiposity and the risk of obesity-related complications [11]. In this context, alternative biomarkers based on regional measurements such as waist circumference (WC), hip circumference (HC), waist-to-hip ratio (WHR), waist-to-height ratio (WHR) that more accurately indicates body fat distribution have been established to predict central adiposity. WC and HC have been identified as the strong risk predictors for hypertension, diabetes, high cholesterol, arthritis, coronary heart disease and cancer [12]. However, the potential associations of obesity-related markers with semen quality are unclear. A meta-analysis study on the role of obesity based on BMI on semen quality showed that overweight and/or obesity are associated with poor semen quality parameters [7]. However, semen parameters have been associated with central obesity indices, including WC, HC, WHpR and WHtR, but not with BMI [13]. On the other hand, others have suggested that BMI and central obesity indices fail to predict semen quality [14, 15].

In recent years, excessive fat accumulation around the neck, a measurement of upper body subcutaneous fat distribution, has been proposed as an indicator of central adiposity because it resembles visceral fat more than subcutaneous fat [16]. Indeed, a large neck circumference (NC) has been uniquely associated with an increased risk of several health problems, such as cardiovascular disease, hyperinsulinemia, metabolic syndrome, and obstructive sleep apnea, independent of BMI and other central obesity indices [17–19]. Moreover, NC may be a useful tool for screening excess body fat, as it is an easy-to-apply, non-invasive, low-cost method and a more stable measure than WC. However, to date, the association of NC with semen parameters and its potential role in predicting male infertility has not been studied.

The first aim of this study was to investigate the potential role of obesity defined by BMI and central obesity indices, which remains unclear today, in male infertility. The second aim of the study was to compare the potentials of BMI and central obesity indices in predicting poor semen quality and to determine the possible superiority of central obesity over BMI. The third aim of the study was to demonstrate the predictive potential of NC, a noval central obesity index, for semen quality. To achieve all these aims, the predictive potentials of obesity indices for poor semen parameters were evaluated by Receiver Operating Characteristics (ROC) curve analysis in a large patient population. Furthermore, the associations of BMI and central obesity indices with semen parameters were evaluated by binary logistic regression analysis, after adjusting for potential confounding factors.

## Subjects and Methods

### Study Population

The present study is a cross-sectional study and the study population consisted of a total of 4739 male participants aged 17–55 who applied to Selcuk University Hospital Andrology Laboratory for standard semen analysis between 2017–2022 years. The study was approved by the Selcuk University Faculty of Medicine Ethics Committee. Ages, anthropometric obesity measurements, and semen analysis parameters of all participants were recorded. NC measurements were obtained from 3665 of the participants. Sperm viability test were performed on a total of 4438 semen samples. The inclusion criteria of the study were all patients between the ages of 17–55 years and without testicular tumors, clinical varicocele, genital tract infection, and azoospermia.

### Semen Analysis

Semen parameters were evaluated according to WHO 2010 guideline [20] and included sperm concentration, total sperm count, total sperm motility, progressive sperm motility, rapid progressive sperm motility and sperm vitality. Semen variables were divided to the categories according to the reference values for semen parameters provided by the WHO 2010 criteria; sperm concentration: ≥ 15 M/ml and < 15 M/ml, total count: ≥ 39 M and < 39 M, total motility ≥ 40% and < 40%, progressive motility ≥ 32% and < 32%, rapid progressive motility ≥ 5% and < 5%, vitality ≥ 58% and < 58%.

Semen samples were collected by masturbation after a period of 2–7 days of sexual abstinence. The volumes of the semen samples were measured and recorded. Semen samples were left to liquefy for 30–60 min at 37 °C. After liquefaction, sperm concentration and motility characteristics were assessed with a Makler counting chamber under a phase contrast microscope at × 200 magnification. Sperm concentration was determined by randomly scanning at least thirty of the 100 squares in the microscope field. The total sperm count was calculated by multiplying the concentration by the semen volume. Sperm motility was classified as total sperm motility (WHO class A + B + C), progressive motility (WHO class A + B) and rapid progressive motility (WHO class A). Sperm count and motility assessments were performed in duplicate and compared, and when the difference between them exceeded the acceptance limits, the assessments were repeated.

Sperm viability test was performed with Eosin-Nigrosin staining. Semen samples stained with Eosin-Nigrosin were evaluated for viability under an Olympus CX21 microscope at × 400 magnification by examining at least 200 spermatozoa cells per semen sample. Stained cells indicating a cell membrane permeable to the stain were considered non-viable sperm cells, while unstained transparent cells whose cell membrane was impermeable were considered viable sperm cells. Percentage of sperm viability was calculated by dividing the viable cells by the total cells.

### Anthropometric Measurements

As the anthropometric measurements of obesity, BMI, WC, HC, WHpR, WHtR and NC were examined. Participants' height and weight were measured in standing and upright position, without shoes and heavy outer clothing. BMI was calculated as weight in kilograms divided by height in meters squared. WC was measured at the midpoint between the lowest costal margin and the iliac crest, and HC was measured at the widest point around the greater trochanter. WHpR was calculated as waist circumference divided by hip circumference, and WHtR was calculated as waist circumference divided by height. NC was measured perpendicular to the neck axis, above the laryngeal prominence. Obesity was classified according to the WHO guideline (2000) [9]; normal weight: BMI < 25 and overweight/obese: BMI ≥ 25. Central obesity indices were divided into two categories, according to the cutoff points we determined for low semen parameters by ROC curve analysis.

### Statistical Analysis

All statistical analysis was performed using *R* version 4.1.2 (The R Foundation for Statistical Computing. Vienna. Austria; https://www.r-project.org). The Kolmogorov–Smirnov test was applied to determine whether the analyzed parameters were normally distributed. The differences of age and anthropometric obesity measurements between the categories of semen parameters were compared using Mann Whitney U test. Descriptive values were given as mean ± standard deviation and median. *P* value < 0.05 was considered statistically significant.

ROC curve analysis was applied to evaluate the predictive potential of obesity indices for low semen parameters. The area under the curve (AUROC) was calculated with 95% confidence intervals. The optimum cut-off values of obesity indices obtained by Youden index. The determined cut-off values, sensitivity and specificity were calculated with 95% confidence intervals. *P* value < 0.05 was considered statistically significant.

The associations between the categories of semen parameters and obesity indices were analyzed by binary logistic regression analysis after adjusting confounding factors. The associations of BMI with semen parameters were assessed after adjusting for age and smoking status. The associations of central obesity indices with semen parameters were evaluated after adjusting for age, smoking status and BMI. Odds ratios were calculated with 95% confidence intervals. *P* value < 0.10 was considered statistically significant.

## Results

### The Associations of Age and Anthropometric Obesity Indices with Semen Parameters

The average age of the participants included in the study was 29.6 years, ranged from 17–55. Age, anthropometric obesity indices and semen parameters of participants are summarized in Table 1. Tables 2 and 3 demonstrate age and obesity indices of participants according to the categories of semen parameters. Age, BMI and all central obesity indices showed significantly differences between the categories of low and normal semen parameters for all semen parameters; the participants with lower parameters had higher values than those with normal parameters.

**Table 1:** Age, anthropometric obesity indices and semen parameters of male participants

Variable	N	Mean ± SD	Median
Age (years)	4739	29,64 ± 6,67	29
BMI (kg/m^2^)	4739	25,93 ± 4,37	25,60
WC (cm)	4739	95,64 ± 11,45	95
HC (cm)	4739	104,08 ± 9,17	104
WHpR	4739	0,92 ± 0,06	0,92
WHtR	4739	0,54 ± 0,06	0,54
NC (cm)	3665	39,99 ± 3,20	40
Semen Volume (ml)	4739	3,32 ± 1,49	3
Sperm Concentration (10^6^ per ml)	4739	32,99 ± 33,06	23
Total Sperm Count (10^6^ per ejaculate)	4739	108,09 ± 120,08	70
Total Sperm Motility (%)	4739	57,55 ± 24,09	65
Progressive Sperm Motility (%)	4739	43,75 ± 20,49	48
Rapid Progressive Sperm Motility (%)	4739	4,89 ± 5,27	3
Sperm Vitality (%)	4438	41,65 ± 29,79	45

**Table 2:** Age and anthropometric obesity indices of male participants according to the categories of sperm concentration, total sperm count and sperm vitality

		**Age (years)**	**BMI (kg/m**^**2**^**)**	**WC (cm)**	**HC (cm)**	**WHpR**	**WHtR**	**NC (cm)**
**Sperm Concentration**	** ≥ 15 M/ml (N = 2872)**	29,12 ± 6,66 (28)	25,69 ± 4,32 (25,4)	94,75 ± 11,07 (94)	103,38 ± 9,0 (102)	0,91 ± 0,06 (0,92)	0,54 ± 0,06 (0,54)	39,89 ± 3,1 (40)
	** < 15 M/ml (N = 1867)**	30,45 ± 6,66 (30)	26,28 ± 4,40 (26)	97,03 ± 11,87 (97)	105,16 ± 9,32 (104)	0,92 ± 0,06 (0,92)	0,55 ± 0,07 (0,55)	40,13 ± 3,34 (40)
***p*-value**		** < .001**	** < .001**	** < .001**	** < .001**	**0.002**	** < .001**	**0.05**
**Sperm Total Count**	** ≥ 39 M (N = 2928)**	29,08 ± 6,59 (28)	25,65 ± 4,29 (25,34)	94,63 ± 11,07 (94)	103,39 ± 8,96 (102)	0,91 ± 0,06 (0,92)	0,54 ± 0,06 (0,53)	39,87 ± 3,1 (40)
	** < 39 M (N = 1811)**	30,55 ± 6,69 (30)	26,37 ± 4,55 (26,02)	97,28 ± 11,85 (97)	105,2 ± 9,41 (104)	0,92 ± 0,06 (0,92)	0,55 ± 0,06 (0,55)	40,16 ± 3,34 (40)
***p*-value**		** < .001**	** < .001**	** < .001**	** < .001**	** < .001**	** < .001**	**0.017**
**Sperm Vitality**	** ≥ %58 (N = 3363)**	29,1 ± 6,55 (29)	25,68 ± 4,23 (25,38)	95,0 ± 11,02 (94)	103,47 ± 8,9 (103)	0,92 ± 0,06 (0,92)	0,54 ± 0,06 (0,54)	39,97 ± 3,15 (40)
	** < %58 (N = 1075)**	31,32 ± 6,69 (31)	26,56 ± 4,33 (26,23)	97,71 ± 11,82 (97)	105,14 ± 9,15 (104)	0,93 ± 0,06 (0,93)	0,56 ± 0,07 (0,55)	40,28 ± 3,38 (40)
***p*-value**		** < .001**	** < .001**	** < .001**	** < .001**	** < .001**	** < .001**	**0.018**

Values are expressed mean ± standard deviation (median)*. P*-value < 0.05 is considered statistically significant. Sperm concentration ≥ 15 M/ml vs. < 15 M/ml: N = 2159; 1506, sperm total count ≥ 39 M vs. < 39 M: N = 2214; 1451, sperm vitality ≥ %58 vs. < %58: N = 2571; 798 for NC

**Table 3:** Age and anthropometric obesity indices of male participants according to the categories of sperm motility parameters

		**Age (years)**	**BMI (kg/m**^**2**^**)**	**WC (cm)**	**HC (cm)**	**WHpR**	**WHtR**	**NC (cm)**
**Sperm Total Motility**	** ≥ %40 (N = 3966)**	29,3 ± 6,6 (29)	25,78 ± 4,38 (25,46)	95,16 ± 11,28 (94)	103,78 ± 9,18 (103)	0,92 ± 0,06 (0,92)	0,54 ± 0,06 (0,54)	39,91 ± 3,17 (40)
	** < %40 (N = 773)**	31,43 ± 6,7 (31)	26,63 ± 4,22 (26,36)	98,15 ± 11,96 (98)	105,63 ± 8,97 (105)	0,93 ± 0,06 (0,93)	0,56 ± 0,06 (0,56)	40,39 ± 3,32 (41)
***p*-value**		** < .001**	** < .001**	** < .001**	** < .001**	** < .001**	** < .001**	**0.001**
**Sperm Progressive Motility**	** ≥ %32 (N = 3694)**	29,16 ± 6,58 (29)	25,73 ± 4,37 (25,39)	95,05 ± 11,29 (94)	103,7 ± 9,17 (103)	0,91 ± 0,06 (0,92)	0,54 ± 0,06 (0,54)	39,91 ± 3,17 (40)
	** < %32 (N = 1045)**	31,37 ± 6,69 (31)	26,59 ± 4,27 (26,29)	97,75 ± 11,77 (97)	105,44 ± 9,06 (105)	0,92 ± 0,06 (0,93)	0,56 ± 0,06 (0,55)	40,27 ± 3,3 (40)
***p*-value**		** < .001**	** < .001**	** < .001**	** < .001**	** < .001**	** < .001**	**0.005**
**Sperm Rapid Progressive Motility**	** ≥ %25 (N = 1900)**	28,99 ± 6,4 (28)	25,77 ± 4,54 (25,35)	94,22 ± 11,57 (94)	103,84 ± 9,56 (103)	0,91 ± 0,06 (0,91)	0,53 ± 0,07 (0,53)	39,39 ± 3,0 (39)
	** < %25 (N = 2839)**	30,01 ± 6,79 (29)	26,04 ± 4,24 (25,71)	96,6 ± 11,24 (96)	104,26 ± 8,89 (104)	0,92 ± 0,06 (0,93)	0,55 ± 0,06 (0,55)	40,35 ± 3,26 (41)
***p*-value**		** < .001**	**0.004**	** < .001**	**0.05**	** < .001**	** < .001**	** < .001**

Values are expressed as the mean ± standard deviation (median)*. P*-value < 0.05 is considered statistically significant. Sperm total motility ≥ %40 vs. < %40: N = 3070; 595, sperm progressive motility ≥ %32 vs. < %32: N = 2873; 792, sperm rapid progressive motility ≥ %25 vs. < %25: N = 1373; 2292 for NC

### The Predictive Potentials of Anthropometric Obesity Indices for Low Semen Parameters

Table 4 demonstrates the predictive potentials of anthropometric obesity indices for low semen parameters, analyzed by ROC curve. BMI, WC, HC, WHtR and NC could predict low sperm concentration, but WHpR could not. AUCs of BMI, WC, HC, WHtR and NC for low sperm concentration were 0.534 (*p* = 0.001), 0.539 (*p* < 0.001), 0.549 (*p* < 0.001), 0.535 (*p* < 0.001), 0.518 (*p* = 0.05), respectively. The cut-off values of BMI, WC, HC, WHtR and NC for sperm concentration were 25.71, 96.5, 104.5, 0.54 and 40.5 with a sensitivity/specificity of 52.4/52.6%, 52.5/53%, 52.2/56.6%, 52.4/52.6% and 46.4/55.4% respectively. All obesity indices were could predict low sperm total count, total motility, progressive motility and vitality. AUCs of BMI, WC, HC, WHpR, WHtR and NC for low sperm total count were 0.535 (*p* < 0.001), 0.544 (*p* < 0.001), 0.544 (*p* < 0.001), 0.523 (*p* = 0.02), 0.545 (*p* < 0.001), 0.523 (*p* = 0.017), respectively. The cut-off values of BMI, WC, HC, WHpR, WHtR and NC for total count were 25.71, 96.5, 104.5, 0.92, 0.54 and 40.5 with a sensitivity/specificity of 52.3/52.5%, 52.2/53.3%, 51.9/56.2%, 51.1/51.2%, 53.2/53.3%, 47.8/56.2%, respectively. AUCs of BMI, WC, HC, WHpR, WHtR and NC for low sperm total motility were 0.560 (*p* < 0.001), 0.563 *(p* < 0.001), 0.557 (*p* < 0.001), 0.544 (*p* = 0.001), 0.569 (*p* < 0.001), 0.542 (*p* = 0.001), respectively. The cut-off values of BMI, WC, HC, WHpR, WHtR and NC for total motility were 25.95, 97.5, 104.5, 0.92, 0.55 and 40.5 with a sensitivity/specificity of 54.5/55%, 52.5/58.6%, 53.5/54.2%, 52.2/53.1%, 55.2/55.7%, 50.3/55.6%, respectively. AUCs of BMI, WC, HC, WHpR, WHtR and NC for low sperm progressive motility were 0.559 (*p* < 0.001), 0.563 (*p* < 0.001), 0.559 (*p* < 0.001), 0.535 (*p* = 0.002), 0.570 (*p* < 0.001), 0.532 (*p* = 0.005), respectively. The cut-off values of BMI, WC, HC, WHpR, WHtR and NC for progressive motility were 25.93, 97.5, 104.5, 0.92, 0.55 and 40.5 with a sensitivity/specificity of 54.6/54.7%, 52.2/59.3%, 54/54.9%, 51.2/53.2%, 55.4/55.5%, 48.9/55.6%, respectively. AUCs of BMI, WC, HC, WHpR, WHtR and NC for low sperm vitality were 0.562 (*p* < 0.001), 0.565 (*p* < 0.001), 0.553 (*p* < 0.001), 0.546 (*p* < 0.001), 0.572 (*p* < 0.001), 0.528 (*p* = 0.017), respectively. The cut-off values of BMI, WC, HC, WHpR, WHtR and NC for sperm vitality were 25.95, 97.5, 104.5, 0.92, 0.55 and 40.5 with a sensitivity/specificity of 55.4/55.5%, 52.8/59.2%, 51.6/55.9%, 51.9/53%, 55.7/56%, 49.2/54.6%, respectively. WC, WHpR, WHtR and NC were could predict low rapid progressive sperm motility, but BMI and HC could not. AUCs of WC, WHpR, WHtR and NC for low sperm rapid progressive motility were 0.561 (*p* < 0.001), 0.605 (*p* < 0.001), 0.568 (*p* < 0.001), 0.587 (*p* < 0.001), respectively. The cut-off values of WC, WHpR, WHtR and NC for rapid progressive motility were 96.5, 0.92, 0.54 and 40.5 with a sensitivity/specificity of 52.8/56.5%, 57.4/58.6%, 54.4/54.7%, 51.6/65.1%, respectively.

**Table 4:** The predictive potentials of anthropometric obesity indices for low semen parameters

		ROC Curve Analysis			Statistical Diagnostic Measures (95% CIs)	
		AUC (95% CI)	***p***-value	Cut-off	Sensitivity	Specificity
**Sperm Concentration**	BMI	0.534 (0.515– 0.552)	**0.001**	> 25.71	52.4	52.6
	WC	0.539 (0.520 – 0.558)	** < .001**	> 96.5	52.5	53.0
	HC	0.549 (0.530– 0.568)	** < .001**	> 104.5	52.2	56.6
	WHpR	0.511 (0.492 – 0.530)	> 0.05	–	–	–
	WHtR	0.535 (0.516 – 0.554)	** < .001**	> 0.54	52.4	52.6
	NC	0.518 (0.499 – 0.537)	**0.05**	> 40.5	46.4	55.4
**Sperm Total Count**	BMI	0.535 (0.516 – 0.554)	** < .001**	> 25.71	52.3	52.5
	WC	0.544 (0.525 – 0.563)	** < .001**	> 96.5	53.2	53.3
	HC	0.544 (0.525 – 0.564)	** < .001**	> 104.5	51.9	56.2
	WHpR	0.523 (0.504 – 0.542)	**0.02**	> 0.92	51.1	51.2
	WHtR	0.545 (0.526 – 0.564)	** < .001**	> 0.54	53.2	53.3
	NC	0.523 (0.504 – 0.543)	**0.017**	> 40.5	47.8	56.2
**Sperm Total Motility**	BMI	0.560 (0.535 – 0.584)	** < .001**	> 25.95	54.5	55.0
	WC	0.563 (0.537 – 0.589)	** < .001**	> 97.5	52.5	58.6
	HC	0.557 (0.533– 0.582)	** < .001**	> 104.5	53.5	54.2
	WHpR	0.544 (0.518 – 0.570)	**.001**	> 0.92	52.2	53.1
	WHtR	0.569 (0.543 – 0.594)	** < .001**	> 0.55	55.2	55.7
	NC	0.542 (0.516 – 0.567)	**.001**	> 40.5	50.3	55.6
**Sperm Progressive Motility**	BMI	0.559 (0.537 – 0.581)	** < .001**	> 25.93	54.6	54.7
	WC	0.563 (0.541 – 0.586)	** < .001**	> 97.5	52.2	59.3
	HC	0.559 (0.537 – 0.581)	** < .001**	> 104.5	54.0	54.9
	WHpR	0.535 (0.512 – 0.559)	**.002**	> 0.92	51.2	53.2
	WHtR	0.570 (0.547 – 0.593)	** < .001**	> 0.55	55.4	55.5
	NC	0.532 (0.509 – 0.555)	**.005**	> 40.5	48.9	55.6
**Sperm Rapid Progressive Motility**	BMI	0.513 (0.494 – 0.532)	> 0.05	-	-	-
	WC	0.561 (0.541 – 0.580)	** < .001**	> 96.5	52.8	56.5
	HC	0.500 (0.480 – 0.519)	> 0.05	-	-	-
	WHpR	0.605 (0.586 – 0.624)	** < .001**	> 0.92	57.4	58.6
	WHtR	0.568 (0.549 – 0.588)	** < .001**	> 0.54	54.4	54.7
	NC	0.587 (0.568 – 0.606)	** < .001**	> 40.5	51.6	65.1
**Sperm Vitality**	BMI	0.562 (0.540 – 0.585)	** < .001**	> 25.95	55.4	55.5
	WC	0.565 (0.542 – 0.588)	** < .001**	> 97.5	52.8	59.6
	HC	0.553 (0.530 – 0.576)	** < .001**	> 104.5	51.6	55.9
	WHpR	0.546 (0.523 – 0.569)	** < .001**	> 0.92	52.9	53.0
	WHtR	0.572 (0.549 – 0.595)	** < .001**	> 0.55	55.7	56.0
	NC	0.528 (0.505 – 0.551)	**.017**	> 40.5	49.2	54.6

*P*-value < 0.05 is considered statistically significant

HC had relatively higher AUC values with higher specificity for sperm numerical parameters. WHtR and WC had relatively higher AUC values for sperm vitality and motility parameters, except for rapid progressive motility, WHtR had higher sensitivity and WC had higher specificity. For sperm rapid progressive motility, higher AUC values of WHpR and NC were noted, with higher sensitivity of WHpR and higher specificity of NC.

### The Associations of Anthropometric Obesity Indices with Semen Parameters after Adjusting for Potential Confounding Factors

Binary logistic regression analysis showed that, after adjusting for age and smoking status, higher BMI was associated with lower semen parameters, except sperm rapid progressive motility (Table 5). When adjusting for age, smoking status and additional BMI, sperm concentration was found to be associated with WC and HC, sperm total count, total motility and progressive motility with WC, HC and WHtR, sperm rapid progressive motility with WC, WHpR, WHtR and NC and sperm vitality with WC and WHtR (Table 5). The patients with lower sperm concentration (< 15 M/ml) had a WC greater than 96.5 cm (OR: 1.23, *p* = 0.008) and a HC greater than104.5 cm (OR: 1.45, *p* < 0.001). Lower sperm total count (< 39 M) was associated with a WC greater than 96.5 cm (OR: 1.26, *p* = 0.003), a HC greater than104.5 cm (OR: 1.34, *p* < 0.001) and a WHtR greater than 0.54 (OR: 1.17, *p* = 0.04). Lower sperm total motility (< 40%) was associated with a WC greater than 97.5 cm (OR: 1.40, *p* = 0.001), a HC greater than104.5 cm (OR: 1.18, *p* = 0.089) and a WHtR greater than 0.55 (OR: 1.45, *p* < 0.001). The patients with lower sperm progressive motility (< 32%) had a WC greater than 97.5 cm (OR: 1.33, *p* = 0.002), a HC greater than104.5 cm (OR: 1.16, *p* = 0.087) and a WHtR greater than 0.55 (OR: 1.35, *p* = 0.001). The patients with lower sperm rapid progressive motility (< 25%) had a WC greater than 96.5 cm (OR: 1.54, *p* < 0.001), a WHpR greater than 0.92 (OR: 1.71, *p* < 0.001), a WHtR greater than 0.54 (OR: 1.55, *p* < 0.001) and a NC greater than 40.5 cm (OR: 2.37, p < 0.001). Lower sperm vitality (< 58%) was associated with a WC greater than 97.5 cm (OR: 1.37, *p* = 0.001) and a WHtR greater than 0.55 (OR: 1.34, *p* = 0.002).

**Table 5:** The associations of anthropometric obesity indices with semen parameters after adjustment potential confounding factors

Semen Parameters	Anthropometric Obesity Indices	OR	95% Confidence Intervals	*p*-value
**Sperm Concentration (≥ 15 M/ml vs. < 15 M/ml)**	BMI	1.020	1.006 – 1.034	0.005*
	WC	1.229	1.056 – 1.430	**.008**
	HC	1.453	1.256 – 1.681	** < .001**
	WHpR	1.004	0.888 – 1.137	> .10
	WHtR	1.121	0.963 – 1.304	> .10
	NC	0.940	0.809 – 1.091	> .10
**Sperm Total Count (≥ 39 M vs. < 39 M)**	BMI	1.025	1.011 – 1.039	< .001*
	WC	1.259	1.081 – 1.466	**.003**
	HC	1.343	1.160 – 1.554	** < .001**
	WHpR	1.047	0.925 – 1.186	> .10
	WHtR	1.173	1.008 – 1.367	**.04**
	NC	1.041	0.896 – 1.210	> .10
**Sperm Total Motility (≥ %40 vs. < %40)**	BMI	1.022	1.004 – 1.040	0.017*
	WC	1.396	1.143 – 1.707	**.001**
	HC	1.183	0.975 – 1.435	**0.089**
	WHpR	1.072	0.906 – 1.268	> .10
	WHtR	1.451	1.182 – 1.781	** < .001**
	NC	1.046	0.854 – 1.281	> .10
**Sperm Progressive Motility (≥ %32 vs. < %32)**	BMI	1.023	1.007 – 1.040	0.006*
	WC	1.331	1.112 – 1.593	**.002**
	HC	1.163	0.978 – 1.383	**0.087**
	WHpR	1.016	0.874 – 1.180	> .10
	WHtR	1.347	1.122– 1.618	**.001**
	NC	0.968	0.806 – 1.161	> .10
**Sperm Rapid Progressive Motility (≥ %25 vs. < %25)**	BMI	1.003	0.989 – 1.017	> .10*
	WC	1.542	1.322 – 1.798	** < .001**
	HC	0.887	0.763 – 1.030	> .10
	WHpR	1.715	1.510 – 1.948	** < .001**
	WHtR	1.551	1.326 – 1.813	** < .001**
	NC	2.373	2.020 – 2.787	** < .001**
**Sperm Vitality (≥ %58 vs. < %58)**	BMI	1.025	1.009 – 1.043	0.003*
	WC	1.371	1.143 – 1.644	**.001**
	HC	1.078	0.905 – 1.285	> .10
	WHpR	1.038	0.893 – 1.206	> .10
	WHtR	1.343	1.116– 1.616	**.002**
	NC	0.899	0.747 – 1.081	> .10

*P *values were adjusted for age, smoking status and BMI. *P values were adjusted for age and smoking status. *P*-value < 0.10 is considered statistically significant.

## Discussion

Currently, the association between obesity and semen quality remains unclear. Most studies to date have focused on the role of obesity defined based on BMI on semen quality. Recent meta-analysis studies have shown that obesity is associated with different infertility markers. The meta-analysis by Salas-Huetos et al. have proven that obesity was associated with poor semen quality, including semen volume, sperm concentration, total count, total motility, progressive motility, and normal morphology [7]. The meta-analysis by Zhong et al. highlighted the predictive value of BMI for low sperm concentration, total count and progressive motility parameters [21]. The meta-analysis by Guo et al. documented that BMI was negatively correlated with sperm concentration and total count, but not with sperm total or progressive motility [22]. On the other hand, no link has been found between BMI and poor semen quality [13–15]. Our study showed that BMI was associated with semen parameters, except sperm rapid progressive motility and has predictive potential for the same parameters. Moreover, it was observed that these associations persisted even adjusted for age and smoking status. The association of BMI with most infertility parameters suggests that it may be a predictor of poor semen quality and male infertility. However, in our study, it is noteworthy that central obesity indices had stronger associations, especially with certain infertility parameters, than BMI. WC exhibited stronger associations with all semen parameters than BMI, after adjusting for the same confounding factors. WHtR had strong associations with semen parameters, except sperm concentration and was superior to BMI for associated semen parameters. WHpR and NC were associated only with sperm rapid progressive motility and were superior to BMI for this parameter. HC was associated with semen parameters, except sperm rapid progressive motility and vitality and was superior to BMI for sperm count parameters. All central obesity indices showed significant positive correlations with age and BMI. Furthermore, after further adjustment for BMI, the strongest associations were found between WC and WHtR and sperm vitality and motility parameters (including rapid progressive motility), WHpR and NC and rapid progressive sperm motility, and HC and sperm count parameters. Therefore, our results suggest that central obesity could predict poor semen quality, independently and more strongly than BMI.

The association of different obesity markers with certain infertility parameters suggests that body fat distribution may have specific roles on sperm functionality, resulting in different sperm parameters may be affected. After spermatozoa are produced in the testicles through the process of spermatogenesis, they acquire progressive motility characteristics and fertilization abilities during their storage and transfer in the epididymal duct, known as the epididymal maturation process. Spermatozoa that progress through the genital tract receive secretions from the post-testicular ducts and, to a large extent, from the accessory glands, which further influence their motility. Therefore, according to our study results, it may be possible that fat accumulation around the waist (and, by extension, WHtR and WHpR) and around the neck may impair particularly post-testicular functions including epididymal maturation process, resulting in deterioration in sperm motility parameters and semen volume. Fat accumulation around the hips, which has stronger associations with low sperm count parameters, may affect testicular functions more, leading to deterioration in spermatogenesis. The strong association of rapid progressive motility, a highly specialized function of spermatozoa, with obesity markers beyond markers affecting progressive motility, suggests that specific adipose tissue-related factors, especially NC-related, may play a role in this function. As a result, we believe there are some specific factors associated with hip, waist, and neck fat that may specifically affect different fertility markers.

Although the potential mechanisms underlying the role of excess adiposity on semen quality are not fully understood, several possible mechanisms have been implicated, including alterations in the sex hormone profile, increased scrotal temperature due to excess fat accumulation in the region, induced oxidative stress and apoptosis and increased production of adipokines and pro-inflammatory cytokines, and epigenetic modifications. Visceral adiposity alters hormonal balance in obese men, leading to decreased plasma testosterone levels and increased estradiol levels, which are primarily produced by androgen aromatization in abdominal fat tissues [23]. Many studies have linked low testosterone and high estradiol levels in obese men, along with decreased testosterone/estradiol levels, to poor semen quality [7, 13–15, 21, 24]. Excess visceral fat also causes insulin resistance and increases insulin levels. Elevated insulin levels reduce plasma sex hormone-binding globulin (SHBG) production in the liver, leading to a reduction in testosterone and an increase in estradiol [25]. Adipose tissue also produces a variety of adipokines and proinflammatory cytokines linked to obesity-associated male infertility, including leptin, adiponectin, resistin, ghrelin, TNF-alpha, IL-1β and IL-6 [8, 26, 27]. In obese individuals, increased secretion of leptin maintains a high estrogenic state or directly affects spermatogenesis and androgen production by Leydig cells [23, 27]. Low-grade systemic inflammation, which can accompany obesity, causes high levels of ROS and oxidative stress. Excess ROS levels may cause DNA damage in spermatozoa and disrupt mitochondria and nucleus, resulting in decreased sperm motility and fertilization ability [28].

The stronger association of WC and WHtR with most infertility markers than BMI can be explained by the negative metabolic effects resulting from these hormonal alterations, especially in abdominal fat tissues, which disrupt sperm functions. WC has been more strongly associated with hyperglycemia, hyperinsulinemia, and dyslipidemia than BMI, and suggested to be a useful predictor of the adverse metabolic effects of central adiposity on semen quality [29]. Recent studies have found that WC, HC, WHpR, and WHtR have stronger negative correlations than BMI, which is consistent with our results [13, 24, 30, 31]. WHtR [24] and WHpR [31] have been proposed to be the strongest predictors of sperm progressive motility, with height and WHtR in association with plasma testosterone and insulin levels [24], and WHpR in association with plasma total cholesterol and low-density lipoprotein (LDL) levels [13, 24]. Our study suggests that WHtR and WC are the strongest predictors for total and progressive sperm motility, WHpR and NC are the strongest predictors for rapid progressive motility and HC is the strongest predictor for sperm count parameters. Seminal plasma levels of vaspin, a recently identified adipocytokine, and sperm progressive motility were shown to be important independent determinants of poor sperm DNA integrity [32]. Moreover, in patients with obstructive sleep apnea syndrome, plasma vaspin levels were associated with BMI, WC, WHpR and NC as well as total triglyceride and insulin levels, but not with HC [33]. Therefore, taking into account the results of these previous studies, we think that particularly WHpR and NC-related vaspin and blood lipids may be involved in the rapid progressive motility characteristics of sperm. The lack of association of seminal plasma vaspin levels with HC also supports our finding that HC is weakly associated with sperm motility parameters and not at all with rapid progressive motility. It has also been shown that total sperm count, total and progressive sperm motility was associated with plasminogen activator inhibitor-1 (PAI-1) and visfatin, whereas glucagon-like peptide-1 (GLP-1) was associated with progressive motility only [34]. The association of obesity markers with different adipokines, hormone peptides and lipids, which are probably differentially regulated by body fat distribution, may be the mechanism behind their associations with different functional characteristics of spermatozoa. Another mechanism of testicular dysfunction caused by obesity is heat-induced dys-spermatogenesis. Testicular temperature is high in obese men, and this is mainly caused by excess fat accumulation in areas close to the testicles [23]. This may explain the strong association between a large HC and decreased sperm count parameters, according to our study results.

NC, a novel central obesity index, has been shown to have a unique association with many health conditions, such as cardiovascular disease, hyperinsulinemia, metabolic syndrome, diabetes, and obstructive sleep apnea, independently of BMI and other central adiposity indices [17–19]. In a study conducted in patients with type 2 diabetes in the Turkish population, NC was shown to be associated with WC, systolic blood pressure, triglycerides and high-density lipoprotein (HDL) in men, and the cut-off value for metabolic syndrome was found to be 39 cm [35]. It has been shown that NC, WHpR and WC was strong predictors of insulin resistance in women with polycystic ovary syndrome [36], and that NC even outperformed other anthropometric measurements in predicting metabolic conditions [37]. Interestingly, most of the associations of NC with metabolic conditions were found to be stronger in men than in women, suggesting that there may be additional factors specific to neck fat accumulation in men. The unique association of NC with many health conditions that lead to greater risk of male infertility has led us to question whether NC is also a predictor of male infertility. However, the relationship of NC to semen quality has not been investigated to date. The current study showed that NC could predict all low semen parameters, but, when adjusting for confounding factors, it was only associated with rapid progressive sperm motility more strongly than other indices of obesity. The association between neck fat and metabolic syndrome has been attributed to the excessive release of free fatty acids from upper body subcutaneous fat into plasma by lipolytic activity, which is more harmful than free fatty acids released from lower body subcutaneous fat, leading to oxidative stress and insulin resistance [38, 39]. Lipid peroxidation damage of these substrates by ROS produces genotoxic products that suppress male fertility, especially motility characteristics of sperm. It is therefore understandable that a greater oxidative stress caused by the higher free fatty acids associated with neck fat would further affect sperm motility function. Additionally, previously established neck fat-associated factors such as vaspin [33], triglyceride, LDL, HDL and insulin [33, 35, 36] may also be involved in the impairment of rapid progressive sperm motility. A previous study showed that boys and girls with larger NC had significantly greater fat content in the android region, suggesting that NC is a predictive factor for excess android fat [40]. In our study, poor sperm motility of individuals with a large NC may be related to the excessive android fat content of these individuals. However, the potential mechanisms of the effects of excess neck fat on sperm function need to be further investigated. Limitations of the current study are that the fat distribution was not determined by more precise methods such as computed tomography or MRI. It may be important to evaluate the relationship between the amount of fat in the android region and semen quality.

## Conclusions

The current study demonstrates that obesity, as determined by BMI and central obesity indices, plays an important role in male infertility. Central obesity indices have stronger associations with certain semen parameters independent of BMI, and appear to be superior to BMI in predicting semen quality. WC and WHtR are the strongest predictors for sperm total and progressive motility and vitality parameters, while HC are the strongest predictor for sperm count parameters. NC and WHpR, a novel index of central obesity, are the strongest predictors of sperm rapid progressive motility, which is a highly specialized function of sperm and can further predict fertilization capacity, indicating the importance of these parameters in male infertility. The association of different obesity markers with certain infertility parameters suggests that body fat distribution, which plays a role in the differential regulation of adipokines, hormone peptides and lipids, may have specific roles in sperm functional characteristics, resulting in different sperm parameters may be affected. However, the mechanisms by which obesity impairs male reproductive functions are numerous and complex and further studies are needed to fully elucidate body fat distribution-related factors that may be involved in semen quality.
